# Cellular Uptakes, Biostabilities and Anti-miR-210 Activities of Chiral Arginine-PNAs in Leukaemic K562 Cells

**DOI:** 10.1002/cbic.201100745

**Published:** 2012-05-25

**Authors:** Alex Manicardi, Enrica Fabbri, Tullia Tedeschi, Stefano Sforza, Nicoletta Bianchi, Eleonora Brognara, Roberto Gambari, Rosangela Marchelli, Roberto Corradini

**Affiliations:** a Dipartimento di Chimica Organica e Industriale, Università di ParmaParco Area delle Scienze 17A, 43124 Parma (Italy); b Dipartimento di Biochimica e Biologia Molecolare, Sezione di Biologia Molecolare, Università di FerraraVia Fossato di Mortara 74, 44121 Ferrara (Italy); c Centro di BiotecnologieVia Fossato di Mortara 64, 44121 Ferrara (Italy)

**Keywords:** cell permeation, cellular differentiation, chiral PNA, microRNA, peptide nucleic acids, RNA

## Abstract

A series of 18-mer peptide nucleic acids (PNAs) targeted against micro-RNA miR-210 was synthesised and tested in a cellular system. Unmodified PNAs, R_8_-conjugated PNAs and modified PNAs containing eight arginine residues on the backbone, either as C2-modified (*R*) or C5-modified (*S*) monomers, all with the same sequence, were compared. Two different models were used for the modified PNAs: one with alternated chiral and achiral monomers and one with a stretch of chiral monomers at the N terminus. The melting temperatures of these derivatives were found to be extremely high and 5 m urea was used to assess differences between the different structures. FACS analysis and qRT-PCR on K562 chronic myelogenous leukaemic cells indicated that arginine-conjugated and backbone-modified PNAs display good cellular uptake, with best performances for the C2-modified series. Resistance to enzymatic degradation was found to be higher for the backbone-modified PNAs, thus enhancing the advantage of using these derivatives rather than conjugated PNAs in the cells in serum, and this effect is magnified in the presence of peptidases such as trypsin. Inhibition of miR-210 activity led to changes in the erythroid differentiation pathway, which were more evident in mithramycin-treated cells. Interestingly, the anti-miR activities differed with use of different PNAs, thus suggesting a role of the substituents not only in the cellular uptake, but also in the mechanism of miR recognition and inactivation. This is the first report relating to the use of backbone-modified PNAs as anti-miR agents. The results clearly indicate that backbone-modified PNAs are good candidates for the development of very efficient drugs based on anti-miR activity, due to their enhanced bioavailabilities, and that overall anti-miR performance is a combination of cellular uptake and RNA binding.

## Introduction

**Scheme 1:**
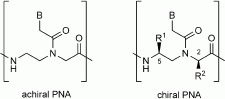
Structures of unmodified achiral PNAs and of C2- and C5-modified arginine PNAs.

Peptide nucleic acids (PNAs, Scheme 1), DNA analogues with polyamidic backbones, are very important tools for the recognition of DNA and have been extensively used for gene modulation. PNAs show even higher affinities for RNA than for DNA.^1^, ^2^ Selective negative modulation of gene expression can be addressed by using a PNA in the antisense strategy, by targeting oncogenes, viruses such as HIV, bacteria, and directing aberrant splicing.^3^ Targeting of chromosomal dsDNA in the so-called anti-gene strategy has also been demonstrated to be a possible approach for down-regulation of specific genes.^4^

MicroRNAs (miRs) are short (19–23 bp) regulatory double-stranded RNAs that modulate gene expression through a mechanism similar to RNA interference: that is, through incorporation of one strand into the miRNA-induced Silencing Complex (miRISC) and suppression of mRNA expression leading to impairment of highly regulated biological functions such as differentiation, cell cycle and apoptosis. Inhibition of miR activity by specific molecules able to bind them by base pairing (antago-miR or anti-miR) has thus been shown to be of great interest in drug development, because it allows restoration of the expression of miR-targeted genes.^5^ Ideally, the anti-miR activity should be accompanied by good cellular uptake, high chemical and enzymatic stability and very high affinity for the target RNA.

PNAs are ideal candidates for targeting miRs because they form very stable PNA:RNA duplexes, which can efficiently disrupt the dsRNA duplex. However, very few works on the use of PNAs as anti-miR agents have so far been reported, and in these PNAs showed the best performances out of a series of oligonucleotide analogues.^6^–^8^

Cellular delivery of PNAs has been improved either by conjugation with carrier molecules or by chemical modification. A very efficient way to induce cellular uptake is to link PNAs to cell-penetrating peptides (CPPs), exploiting the similarity between peptide and PNA solid-phase synthesis. PNAs conjugated with penetratin, transportan and other peptidic carriers have thus been extensively used in antisense research. Conjugation with short cationic peptides such as tetralysine was shown to improve the cellular permeabilities of PNAs greatly^8^, ^9^ and/or to favour dsDNA invasion.^10^ Introduction of positively charged amino acid side chains on the backbone has been shown to favour uptake in several cellular lines^11^, ^12^ and to enhance DNA affinity, thus favouring dsDNA invasion.^13^–^15^

A very efficient delivery system developed by Wender and co-workers is based on arginine oligomers, which have been demonstrated to be better carriers than other peptides containing cationic amino acids, such as poly-Lys or poly-His derivatives.^16^ Oligoarginines were reported to be internalised into mammalian cells through an endocytotic pathway, mediated by binding to cell surface heparan sulfates linked to proteoglycans,^17^ followed by back-transport into the Golgi complex and endoplasmic reticulum.^18^ A modelling study on oligoarginine peptides suggested that not all the arginine side chains are able to interact with the plasma membrane, and a systematic study showed that longer spacing between the arginine side chains could improve the uptake of these carrier peptides, with activity increasing with increasing spacer length, and with optimal values obtained for 6-aminocaproic acid.^19^ In spite of these interesting properties as carriers, antisense oligoarginine–PNA conjugates were reported to have decreased activity in relation to other cationic peptides, which was attributed to their rapid degradation by peptidases.^20^

Substitution at either the C2 or the C5 carbon atoms (Scheme 1) of the PNA backbone with amino acid side chains leads to ambivalent structures with the properties of a DNA or RNA mimic on one hand and of a peptide mimic on the other, thus allowing recognition by specific receptors, as shown very recently by a short PNA mimicking the function of a nuclear localisation signal peptide (NLS).^21^ This strategy can thus be used to obtain PNAs with both peptide properties and RNA-binding capabilities.

Stereochemistry plays a very important role in DNA binding by PNAs. PNA:DNA duplex stability can be modulated by the introduction of functional groups at the C2 and C5 positions of the pseudopeptidic backbone (Scheme 1); we have systematically studied the effect of stereochemistry on induction of helical handedness and DNA binding affinity.^22^, ^23^ Furthermore, the use of chiral PNAs allows higher sequence selectivity to be achieved even for single-mismatch recognition.^24^

MiR-210 is an important microRNA target that has been demonstrated to be associated with mithramycin-mediated induction of erythroid differentiation of leukaemic K562 cells and HbF production in erythroid precursor cells from β-thalassemia patients.^25^, ^26^ In a parallel work we have demonstrated that a PNA 18-mer targeted to miR-210 and linked to a polyarginine chain has the ability to affect cellular differentiation in K562 cells. The regulative pathway of this miR was studied in detail by inhibiting its activity with PNA.^26^ In particular, the regulation of the *raptor* and γ-globin genes by anti-miR-210 PNA was shown.

**Figure 1:**
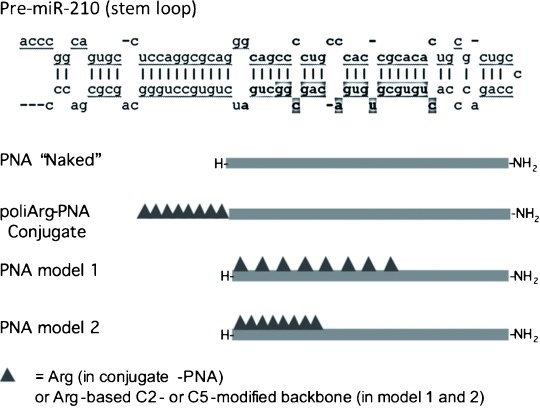
Sequences of pre-miR-210 (upper part of the panel) with sequences corresponding to the mature RISC-associated miR-210 in bold (the guiding strand is indicated); the sequence boxed in grey shows the target region of the PNA used in this study. The bottom part of the panel shows structural models of the investigated PNAs.

Here we describe the use of PNAs incorporating a series of arginine residues in their backbones as polyarginine mimics either at the C2 or at the C5 positions (Figure 1) potentially able to perform specific inhibition of miR-210 in the K562 and other cellular lines, with the aim of developing an optimised model for cellular uptake, biostability and anti-miR activity. The position of the side chain within the PNA monomer and the distribution of the modified monomers along the PNA chain were varied in order to achieve this goal. The results show that these PNAs display uptake similar or even superior to that of the peptide alone and PNA–peptide conjugate, as well as good anti-miR properties. Furthermore, the incorporation of the arginine residues into the PNA backbone was found to be very effective for avoiding enzymatic degradation, thus further increasing the PNA bioavailability in cellular systems even under extreme conditions.

## Results and Discussion

### Model design

Obtained by processing of the *pre*-miR depicted in Figure 1, miR-210 is a 22 bp dsRNA with very important regulatory properties in cellular differentiation. One of the two strands of mature miR-210 (called the guiding strand) is incorporated in the micro-RNA-induced silencing complex (mi-RISC) and has a sequence complementary to the 3′-UTR region of several important mRNA targets (for a complete list see the miRBase site). Because the PNA:RNA duplex is very stable, an 18-mer PNA sequence complementary to the 5′-terminal part of mature miR-210 was gauged to have sufficient affinity to hybridise stably to the miR-210 guiding strand. Analysis of potential binding sites for mRNA targets of this sequence was ruled out by screening for potential sequences in the human transcriptome: only partially complementary sequences (13 bp at maximum) of unrelated genes were found.

Conjugation of the PNA with a polyarginine (R_8_) peptide was shown to be an effective strategy for enhancing cellular permeability allowing anti-miR effects of PNA to be observed,^27^ as demonstrated by good uptake into K562 leukaemic cells and disruption of the miR-210 signalling.

Backbone-modified PNAs bearing positively charged residues have been extensively studied in our group by using lysine and arginine as building blocks; the amino acid side chain has been introduced on C2 or C5 carbons or on both. These models have been shown to display DNA binding abilities strongly dependent on stereochemistry.^28^ Duplexes with complementary DNA containing C2 substitution were found to be much more stable if the PNA was synthesised from d-amino acids (2d-AA), whereas for the C5 substitution the l-enantiomers showed better properties (5l-AA). However, comparison of the miRNA-binding abilities of these two types of PNA has not been reported so far. We thus designed a series of four arginine-based PNAs with different spacing between the side chains and different types of chiral monomers (Figure 1 and Table 1). Overall, each PNA sequence was designed to have the same number of positive charges as in the R_8_-PNA conjugate by incorporating either 2d-Arg or 5l-Arg monomers. Alternating modified monomers were used in model 1 (**PNA3** and **PNA5**), whereas stretches of consecutive modified monomers were used in model 2 (**PNA4** and **PNA6**). The R_8_ peptide modified with fluorescein (**pept-1**) was also produced as a reference for uptake properties in the cellular system used.

**Table 1:** Sequences and models of peptides, PNAs, DNAs and RNAs used in this study. Modified monomers and mismatched bases are in bold.

Compound	Sequence	Modification	Model

Pept-1	Fl-AEEA-R_8_-NH_2_	–	–
PNA1	H-CCGCTGTCACACGCACAG-NH_2_	none	“naked”
PNA2	H-R_8_CCGCTGTCACACGCACAG-NH_2_	none	peptide-conjugated
PNA2-Fl	Flu-AEEA-R_8_-CCGCTGTCACACGCACAG-NH_2_	none	peptide-conjugated
PNA3	**H-cc_5lArg_gc_5lArg_tg_5lArg_tc_5lArg_ac_5lArg_ac_5lArg_gc_5lArg_ac_5lArg_ag-NH_2_**	5l Arg	model 1
PNA3-Fl	Fl-AEEA-C**C_5lArg_**G**C_5lArg_**T**G_5lArg_**T**C_5lArg_**A**C_5lArg_**A**C_5lArg_**G**C_5lArg_**A**C_5lArg_**AG-NH_2_	5l Arg	model 1
PNA4	H-**C_5lArg_C_5lArg_G_5lArg_C_5lArg_T_5lArg_G_5lArg_T_5lArg_C_5lArg_**ACACGCACAG-NH_2_	5l Arg	model 2
PNA4-Fl	Fl-AEEA-**C_5lArg_C_5lArg_G_5lArg_C_5lArg_T_5lArg_G_5lArg_T_5lArg_C_5lArg_**ACACGCACAG-NH_2_	5l Arg	model 2
PNA5	H-c**c_2darg_**g**c_2darg_**t**g_2darg_**t**c_2darg_**a**c_2darg_**a**c_2darg_**g**c_2darg_**a**c_2darg_** AG-NH_2_	2d Arg	model 1
PNA5-Fl	Fl-AEEA-C**C_2dArg_**G**C_2dArg_**T**G_2dArg_**T**C_2dArg_**A**C_2dArg_**A**C_2dArg_**G**C_2dArg_**A**C_2dArg_** AG-NH_2_	2d Arg	model 1
PNA6	H-**C_2dArg_C_2dArg_G_2dArg_C_2dArg_T_2dArg_G_2dArg_T_2dArg_C_2dArg_**ACACGCACAG-NH_2_	2d Arg	model 2
PNA6-Fl	Fl-AEEA-**C_2dArg_C_2dArg_G_2dArg_C_2dArg_T_2dArg_G_2dArg_T_2dArg_C_2dArg_**ACACGCACAG-NH_2_	2d Arg	model 2
DNA1	5′-CTGTGCGTGTGACAGCGG-3′	none	full-match
DNA2	5′-CTGTGCGTGT**T**ACAGCGG-3′	none	mismatched
RNA1	5′-CUGUGCGUGUGACAGCGG-3′	none	full-match
RNA2	5′-CUGUGCGUGU**U**ACAGCGG-3′	none	mismatched

### PNA synthesis

The Boc-protected, arginine-based 2d- or 5l-modified PNA backbone and monomers were synthesised by use of d- or l-Boc-Arg(Tos) as starting material as described previously.^29^ The achiral PNA and the 5l-modified PNAs were synthesised by using preformed monomers. The G- and C-containing modified 5l monomers were available from previous studies,^29^ whereas the 5l monomer bearing the thymine base (**2**) was newly synthesised as described in the Experimental Section by the same strategy.

For the 2d-modified PNAs, the preformed monomers give rise to epimerisation reactions during solid-phase synthesis, as described by us several years ago.^30^ For the synthesis of these derivatives we thus adopted a “sub-monomeric strategy”, which had been shown to give rise to highly stereochemically pure PNAs.^31^ This approach consists of the coupling of the doubly protected *N*-(2-aminoethyl)amino acid (sub-monomer) to the growing chain, followed by α-nitrogen deprotection and coupling of the nucleobase derivative directly on the solid phase. All PNAs synthesised were purified by HPLC and their purities and identities were checked by HPLC-MS (see the Supporting Information).

### Circular dichroism studies

Circular dichroism (CD) spectra of achiral PNA:DNA antiparallel duplexes were systematically described in an early work on PNA-based structures,^32^ and electronic CD of either achiral or chiral PNA has recently been reviewed.^33^

**Figure 2:**
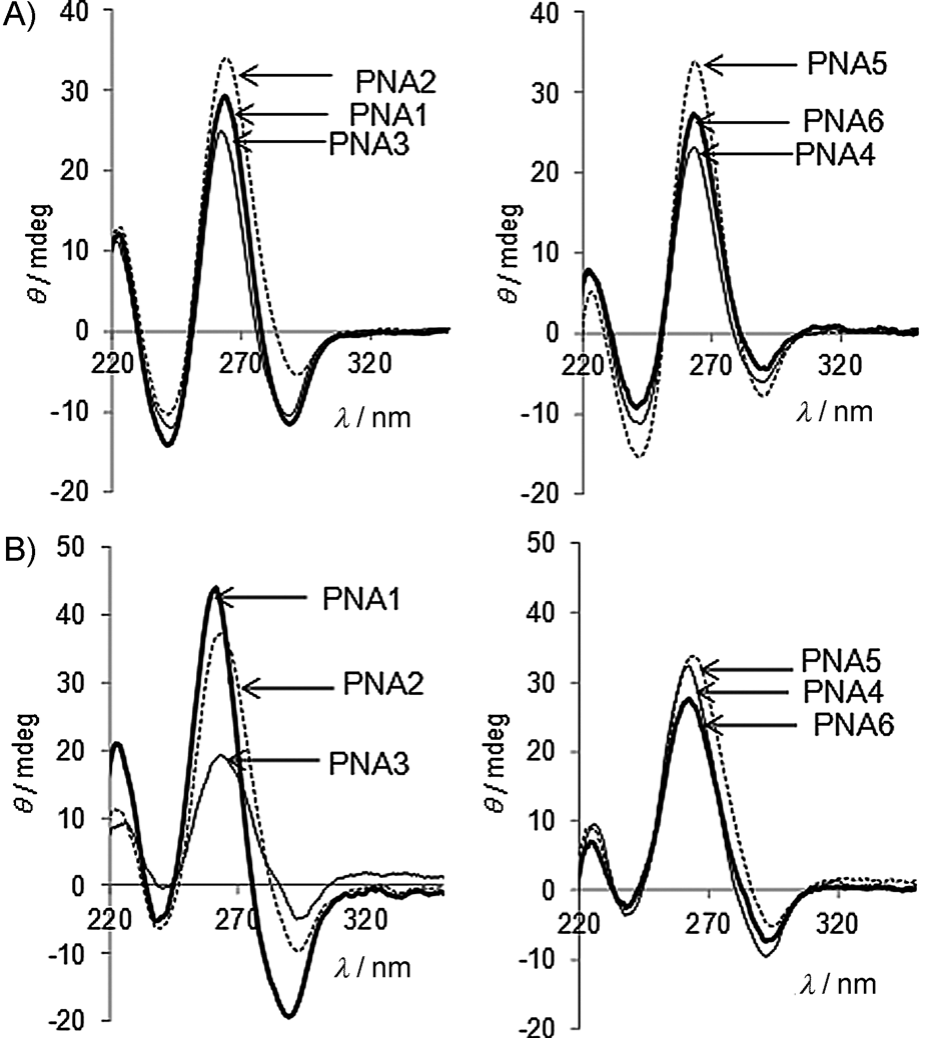
CD spectra of A) PNA:DNA and B) PNA:RNA duplexes with unmodified and modified PNAs.

The PNA:DNA antiparallel duplex formed by an achiral PNA has a complex spectrum as a result of several transitions. Common features of a PNA:DNA spectrum include an intense maximum in the 260–265 nm range, together with a minimum at 240–245 nm (which is much more variable both in sign and intensity); both bands are slightly shifted towards shorter wavelengths in relation to DNA:DNA duplexes of the same sequence. A third band is present in the 280–300 nm region, and can be either positive or negative depending on the sequence. Figure 2 shows spectra of **PNA1**–**6** with antiparallel full-match **DNA1**, and displays very similar features. Therefore, although electrostatic interactions are differently distributed along the PNA chains, the overall conformations of the duplexes are not significantly distorted from that of the achiral, unmodified **PNA1**. Similar behaviour was observed in the presence of antiparallel **RNA1**, corresponding to the target region of miR-210, but in this case more pronounced differences were observed; the most significant differences from the spectrum of the **PNA1**:RNA duplex were observed in the case of **PNA3**, corresponding to 5l-modified PNA with the model 1 structure, and to a lesser extent for **PNA6**, which is 2d-modified PNA by the model 2 scheme. This type of PNA thus seems to induce a preorganisation that prevents the binding to RNA to give a conformation matching that of the unmodified PNA. Interestingly, the same results were obtained when the PNA:DNA or PNA: RNA duplexes were analysed in a solution containing urea (5 m), suggesting that the denaturant is not able to induce their dissociation at room temperature, or even to distort the overall structures of the duplexes. This is in agreement with the extreme thermal stability observed for these duplexes, as described in the next Section.

### Melting temperatures of hybrids with DNA/RNA

In a previous study,^25^ we found that **PNA1** and **PNA2** had melting temperatures that were above 90 °C and therefore not easily determined. The long PNA sequence and the additional electrostatic contribution provided by the arginine peptide (**PNA2**) or arginine-derived monomers (**PNA3**–**6**) induce exceptionally high melting temperatures in water, again exceeding 90 °C. The differences between the analysed structures can therefore be better appreciated under strongly denaturing conditions, such as in buffer containing urea (5 m). Most notably, the *T*_m_ values observed even under strongly denaturing conditions are still very high relative to room temperature, always being in the 70–88 °C range. All of these compounds are thus able to bind strongly both to DNA and to RNA even in this severely unfavourable environment. The highest temperatures observed for PNAs were those of **PNA2**, which is conjugated with the octaarginine peptide, consistently with previous results obtained by us with PNA conjugated with the cationic SV40 NLS peptide.^10^ The melting temperatures of backbone-modified PNA:DNA duplexes under denaturing conditions follow a general trend observed for derivatives of this type in other studies,^23^, ^24^ with 5l-modified (**PNA3** and **PNA4**) or 2d-modified PNA (**PNA5** and **PNA6**) showing higher *T*_m_ values than unmodified **PNA1**. This effect can be attributed to electrostatic contribution to DNA binding, which is present for both 5l- and 2d-Arg-PNAs. The 5l-modified PNAs (**PNA3** and **PNA4**) showed higher *T*_m_ values than the 2d-modified ones (**PNA5** and **PNA6**), consistently with previously reported data, supporting the model of a preferred preorganisation of the 5l-modified backbone,^34^ which is more suitable for formation of the right-handed conformation of the PNA:DNA duplex. Under these strongly denaturing conditions, however, the differences in melting temperatures as a function of backbone modification and stereochemistry were less pronounced than might have been expected from previous studies.

The melting temperatures observed with RNA were higher than those obtained with DNA for unmodified **PNA1**, for the peptide-conjugated **PNA2**, and for the backbone-modified **PNA4** and **PNA5**, whereas in the cases of **PNA3** and **PNA6** they were slightly lower, suggesting that the conformational changes observed in the CD spectrum could negatively affect the abilities of these PNAs to bind RNA, an effect not observed with DNA duplexes.

Sequence selectivity is a very important issue in view of the use of PNAs in cellular systems, because partial hybridisation might occur with other unrelated miRNAs or, most importantly, with mRNA targets. We analysed the sequence selectivities of our PNAs with stricter models (**DNA2** and **RNA2**) involving single base mismatches (G→T or G→U) located in the middle of the target sequences. In each case the sequence selectivity was confirmed by significant lowering of the melting temperature. The ranges of Δ*T*_m_ due to the presence of these single mismatches were 8–21 °C for DNA, with the maximum drop in the case of **PNA4** and the minimum observed in that of **PNA6**. For the latter it should be considered that the mismatch was facing the last chiral monomer, preventing the cooperative effect observed in the case of 2d-modifed highly constrained PNA.^22^

For RNA, the Δ*T*_m_ values due to the presence of the single mismatches were less disperse, varying from 10 to 16, with the best results obtained for **PNA2** and **PNA3** and the worst for **PNA6**. The effects observed for DNA and RNA thus also turned out to be different in the case of mismatch recognition, and prediction either of binding or of selectivity to RNA cannot be inferred from DNA data.

### Uptake and biostability of chiral PNA analogues in K562 cells

The first step necessary to achieve anti-miR activity in cells is effective cellular uptake. We had previously demonstrated that **PNA2**, unlike **PNA1**, was efficiently internalised within leukaemic K562 cells and that this was accompanied by a pronounced anti-miR activity of the former PNA and no activity for the latter.^25^ The K562 cell line has proved to be an excellent experimental system for evaluating the activities against miR-210, because the expression of this miR is increased after mithramycin-induced erythroid differentiation; from the technical point of view, erythroid differentiation can be easily visualised by a benzidine test, whereas treatment with anti-miR-210 agents, including PNAs targeting miR-210, prevents the differentiation process, leading to a different phenotype in respect to MTH-treated cells.^25^

**Figure 3:**
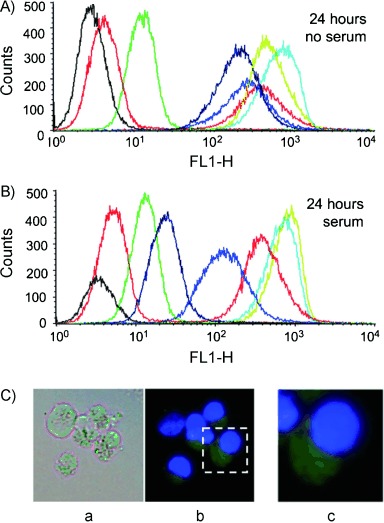
A), B) FACS analysis showing uptake [after incubation (24 h) of K562 cells, either in the absence (A) or in the presence (B) of serum] of fluorescein-labelled **pept-1** (green), **PNA1-Fl** (red), **PNA2-Fl** (dark blue), **PNA3-Fl** (blue), **PNA4-Fl** (black), **PNA5-Fl** (cyan) and **PNA6-Fl** (lime green) molecules. C) Intracellular distribution. a) Cells were cultured for 24 h with **PNA4-Fl** (2 μm) and then analysed by fluorescence microscopy (Nikon Eclipse 80i, 60× magnification). b) and c) Merged analysis of the fluorescence and of the staining of the same cell population with Hoechst 33 258 (selectively staining nuclei).

We therefore studied the uptake of backbone-modified PNAs in the same cellular model and compared the obtained results with those observed in the study of **PNA1** and **PNA2**. K562 cells were incubated in the presence of increasing concentrations of fluorescein-labelled PNAs (**PNA1**–**6-Fl**) for 24 and 48 h and analysed by fluorescence-activated cell sorting analysis (FACS), giving the results shown in Figure 3. Because different PNA structures could be affected by proteases present in serum, leading to different cellular uptakes, these experiments were conducted both in the presence and in the absence of serum. In the absence of serum (Figure 3 A) low internalisation of **PNA1-Fl** was observed (red line), in agreement with previous results. In contrast, the R_8_-conjugated **PNA2-Fl** (blue line) is efficiently internalised. Interestingly, all of the C-2 and C-5 backbone-modified species **PNA3–6-Fl** (both model 1 and model 2) exhibit even higher levels of efficiency of internalisation than **PNA2-Fl**.

In order to determine the distributions of PNAs within target cells, analysis was performed with a fluorescence microscope. A representative example of this analysis is presented in Figure 3 C, showing that the fluorescence of the **PNA6** is mainly cytoplasmatic. Similar results were obtained with all the other chiral PNAs (**PNA3**, **PNA4** and **PNA5**) and also with **PNA2** (data not shown). Low uptake was also found by fluorescence microscopy with **PNA1**.^25^ These data suggest that uptake, rather than simple interactions of PNAs with the cellular membranes, is observed both in the FACS analysis and by microscopy.

Interestingly, in the presence of serum, no decreases in uptake of **PNA5-Fl** or **PNA6-Fl**, and only minor decreases in uptake of **PNA3-Fl** and **PNA4-Fl** were detectable. The most remarkable difference is evident for **PNA2-Fl**, which exhibited decreased uptake by target cells in the presence of serum. This effect might be due to several factors, including aptameric activity of **PNA2-Fl**, leading to binding to serum factors, or to degradation of the peptide part of the molecule by serum proteases. The presence of DNase and protease activity in serum can lead to partial degradation of the carrier polyarginine peptide, whereas the unnatural PNA backbone was reported to be very stable to enzymatic degradation in this medium.^35^ The embedding of the peptide features in the PNA backbone leads to very high stability towards these agents, but with preservation of the recognition elements of the arginine peptide.

**Figure 4:**
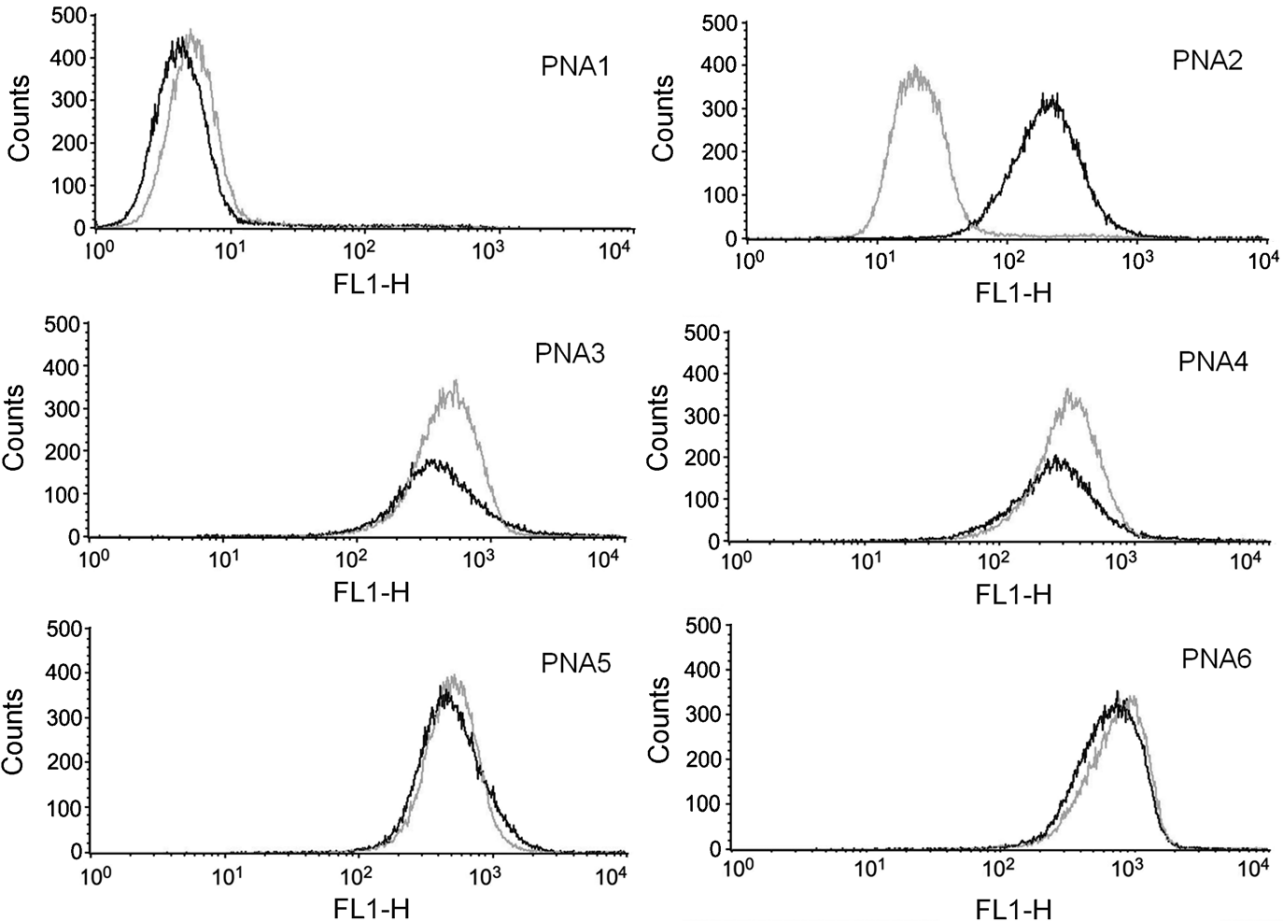
Effects of pre-incubation with trypsin on cellular uptake. FACS analysis showing uptake of fluorescein-labelled **PNA1-Fl**, **PNA2-Fl**, **PNA3-Fl**, **PNA4-Fl**, **PNA5-F**l and **PNA6-Fl** molecules either with (grey) or without (black) pre-incubation with trypsin.

In order to explore the effect of protection against proteases further, pre-incubation of the PNA–peptide conjugate **PNA2-Fl** and the modified PNAs **PNA3-Fl**, **PNA4-Fl**, **PNA5-Fl** and **PNA6-Fl** was performed with trypsin (0.012–0.025 %) before allowing interaction with target K562 cells in the absence of serum (see Figure 4). The obtained results demonstrated that trypsin treatment affects the cellular uptake of **PNA2-Fl**, whereas it does not affect uptake of backbone-modified arginine-PNA. The embedding of arginine residues into the PNA backbone thus allows efficient cellular uptake to be achieved without the risk of degradation even by a basic amino-acid-specific peptidase. In contrast, the carrier peptide of **PNA2-Fl** is to a certain degree an Achilles' heel of the molecule, leading to possible degradation by proteases, an effect worth considering in view of possible developments of PNAs as drugs based on anti-miR activity. The use of backbone-modified PNAs can therefore be viewed as a strategy for delivery of PNAs in an efficient way that is less sensitive to the condition of the medium, as demonstrated by Ly and co-workers in several papers.^12^, ^36^, ^37^

### Chiral PNAs as anti-miR-210 agents

The anti-miR activities of the chiral **PNA3**–**6** were evaluated and compared to that of the previously reported **PNA2**, which had been shown to inhibit miR-210 and also to reduce γ-globin synthesis, thus strongly affecting the differentiation process.

**Figure 5:**
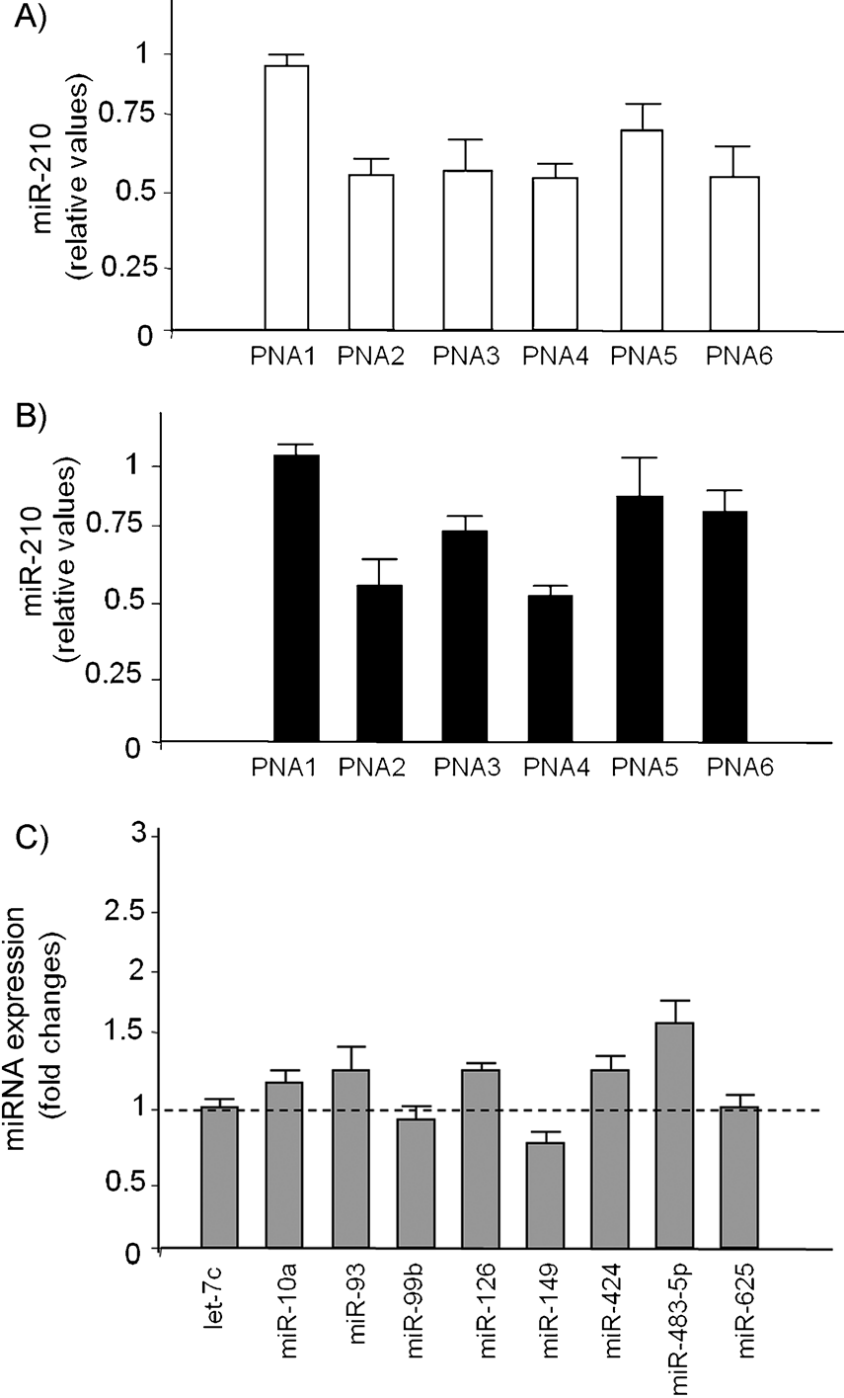
A), B) Effects of treatment with **PNA1**, **PNA2**, **PNA3**, **PNA4**, **PNA5** and **PNA6** molecules on miR-210 contents in cells cultured for 24 h A) in the absence (□), and B) in the presence (▪) of MTH (15 nm). Data are reported as arbitrary units relative to cells treated with **pept-1** (□) or with **pept-1** and MTH (▪). C) Effects of treatment with **PNA4** on miRNA contents in cells cultured for 24 h in the presence of MTH; RT-PCR was performed with a TaqMan MicroRNA Reverse Transcription Kit (Applied Biosystems) for let-7c, miR-10a, miR-93, miR-99b, miR-126, miR-149, miR-424, miR-483-5p and miR-625. Data are reported as arbitrary units relative to untreated cells (average ±SD; *n*=3).

K562 cells were treated with 15 nm mithramycin in the presence of a PNA (2 μm), and after 24 h RNA was isolated and miR-210 was assayed by RT-qPCR analysis. This type of test has been used to evaluate miR content in many studies because it allows the free-miR content to be evaluated. Some authors have reported that degradation of miR also occurs in the presence of oligonucleotide analogues, by a still unknown process, but this results in lower levels both in RT-PCR and in miR activity.^38^ Subsequently, it was shown by Northern blot analysis that in the case of PNAs and LNAs degradation does not occur, but the miR is instead sequestered and made unavailable to its target.^39^ Figure 5 A and B reports the obtained data, showing that inhibition of miR bioavailability was induced both with the modified PNA and with the peptide-conjugated one, whereas in the case of **PNA1** it was unchanged. Stronger inhibition was observed to similar extents for **PNA2**, **PNA3**, **PNA4** and **PNA6**, whereas less efficient inhibition was observed with **PNA5**. This might be related to inhibition of pre-miR maturation or to unavailability of the miR to the qRT-PCR analysis due to strong complexation with the PNA. In either case the bioavailability of the miR for targeting other genes should be reduced.

Differences in miR-210 inhibition became more evident upon treatment of cells with mithramycin, because mithramycin treatment induces expression of miR-210.^26^ Although the effects of various PNAs had been similar in the absence of mithramycin, **PNA2** and **PNA4** gave the best results upon treatment with this drug, whereas **PNA3**, **PNA5** and **PNA6** showed significant inhibition, but to lesser extents. Interestingly, **PNA2** and **PNA4** are those that show the higher *T*_m_ values for the PNA:RNA duplexes (as evaluated under denaturing conditions; see Table 2). Therefore, although all the PNAs considered have very high melting temperatures with complementary RNA, and thus bind quantitatively to complementary RNA in vitro, in the mithramycin-treated cells, producing high levels of miR-210, the inhibition becomes more competitive. The lack of complete inhibition of miR availability, in spite of the very high *T*_m_ values for the PNA:RNA duplexes in vitro (see Table 2), indicates that complete miR-210 inhibition is not achieved in cellular systems (RT-PCR analysis of Figure 5). However, as reported below, the RT-PCR results for miR-210 are associated with downstream inhibitory effects on mRNA, and therefore give a clear indication of anti-miR activity. The anti-miR activity is thus modulated by the type of substitution on the monomer (compare **PNA4** and **PNA6**) and by the distribution of the modified monomers along the chain (compare **PNA3** and **PNA4**). The most efficient model turned out to be the use of C5-substituted monomers with a compact arrangement (model 2), as in **PNA4. PNA2** actually had the same efficiency as **PNA4**, despite its lower cellular uptake; the higher cellular uptake of **PNA4** might be balanced by a higher PNA:RNA stability for **PNA2**, as revealed by *T*_m_ measurements.

**Table 2:** Melting temperatures (*T*_m_) for PNA:DNA and PNA:RNA duplexes, measured by UV absorbance at 260 nm in PBS buffer at pH 7, in the absence or in the presence of urea (5 m). Strand concentration: 5 μm.

PNA^[a]^	DNA1 in water	DNA1 in urea (5 m)	DNA2 in urea (5 m)	RNA1 in water	RNA1 in urea (5 m)	RNA2 in urea (5 m)

PNA1	85	70	58	88	77	62
PNA2	>90	80	68	>90	88	73
PNA3	>90	77	65	>90	76	60 (br)
PNA4	>90	77	56	>90	86	75
PNA5	>90	74	64	>90	78	62
PNA6	>90	77	69	>90	75	65

In view of the stronger effect on miR-210 exhibited by **PNA4**, this PNA was further employed to determine whether the effects on cellular differentiation are comparable to those obtained with **PNA2**. Because **PNA2** had previously been found to inhibit miR-210 selectively in comparison with other miRs,^25^ we studied the sequence-specificity of **PNA4** by measuring its effect on nine other microRNAs expressed in MTHinduced K562 cells; secondly, we compared the activities of **PNA1**, **PNA2** and **PNA4** on γ-globin gene expression and MTH-induced differentiation. Figure 5 C shows the activity of **PNA4** on the hybridisation availability of let-7c, miR-10a, miR-93, miR-99b, miR-126, miR-149, miR-424, miR-494, miR-483-5p and miR-625. Interestingly, no major effects were found, with the exception of inhibition of the hybridisation signal in the case of miR-149. These data demonstrate that **PNA4** does not affect miRs nonspecifically, but displays sequence-selective inhibitory effects.

**Figure 6:**
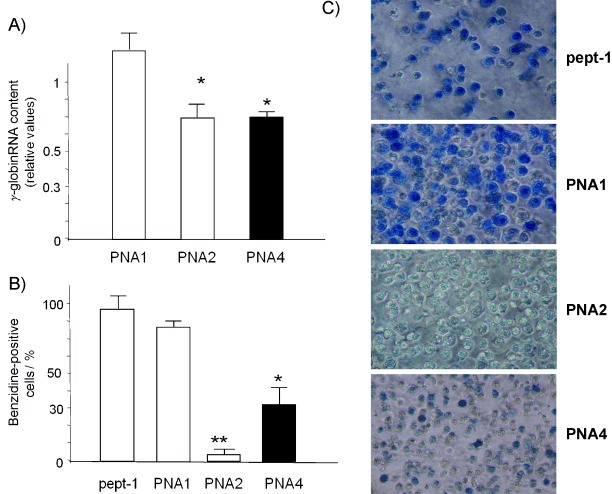
A) Effects of **pept-1**, **PNA1**, **PNA2** and **PNA4** molecules (each 2 μm) on γ-globin content in K562 cell cultures for 72 h with MTH (15 nm). Data are reported as arbitrary units relative to cells treated with pept-1 and MTH (average±SD, *n*=3, *: *p*<0.05, **: *p*<0.01). B), C) Effects of treatment (72 h) with **pept-1**, **PNA1**, **PNA2** and **PNA4** molecules on K562 cells induced to erythroid differentiation by MTH (15 nm): B) summary of three independent experiments, C) a representative experiment showing staining of the cells with benzidine.

It had previously been demonstrated that inhibition of miR-210 either by **PNA2** or by antagomiR treatment causes underexpression of the mithramycin-induced γ-globin gene. The strong inhibition of miR-210 by **PNA2** and **PNA4** might thus result in appreciable reduction of γ-globin gene expression. Fully consistently with this, both **PNA2** and **PNA4** inhibited γ-globin gene expression in mithramycin-treated K562 cells to similar extents, whereas **PNA1** did not show appreciable effects, as determined by RT-qPCR analysis (Figure 6 A); these data were similar to those obtained by multiple daily administrations of antagomiR against miR-210,^25^ but in this case the effect was obtained after a single administration of PNA molecules.

### Effect of PNA4 on erythroid differentiation

In order to evaluate effects on mithramycin-induced cellular differentiation, K562 cells were treated with MTH in the presence of **pept-1**, **PNA1**, **PNA2** and **PNA4** (each 2 μm). The proportions of benzidine-positive cells were determined after 5 days of induction. Figure 6 C shows a representative experiment indicating that both **PNA2** and **PNA4**—unlike **PNA1**, which lacks conjugation to the R_8_ peptide—are able to inhibit erythroid differentiation.

The quantitative analysis is reported in Figure 6 B. In these experiments, significant differences between the effects of **PNA2** and of **PNA4** are observed. The effects of these two PNAs are comparable in the contexts both of miR-210 inhibition and of γ-globin mRNA, so this last result indicates that the conjugation of arginine peptide and the incorporation of the arginine side chains into the PNA structure do not have the same effect. Because these discrepancies cannot be attributed either to differences in the cellular systems used or to interference with the miR-210 pathway, these results might be the consequence of the effect of the R_8_-PNA conjugate on other different pathways, probably mediated by the polyarginine peptide. Although this effect in this case reinforces the anti-differentiating activity of **PNA2**, its consequences are not easily predictable, whereas for the chiral **PNA4** the observed effect is a consequence only of the rationally designed anti-miR activity. The reason for the different bioactivity is unknown; it could also be due to some off-target binding either by **PNA2** further preventing differentiation or by **PNA4**, in this case promoting differentiation. Further studies with a large set of mismatched sequences should help in clarifying this point.

## Conclusions

In this paper we have demonstrated anti-miR activity of backbone-modified PNAs for the first time. New results relating to the cellular uptake of modified PNAs as a function of their structures have been achieved, thereby supporting novel strategies for overcoming one of the most important drawbacks of PNAs and PNA analogues. Overall, the combined good cellular uptakes, higher biostabilities, good anti-miR activities, and reductions in differentiated cells and in γ-globin production make these molecules ideal candidates for the next generation of drugs able to modulate genes. The experimental system (inhibition of miR-210 in K562 cells) was chosen to allow better assessment of the performances of the different types of PNA modification as anti-miR agents. However these results represent a systematic study involving both C2- and C5-modified PNAs with different spacings between modified monomers, and should be extendable to other interesting targets, most notably tumour-associated miR (onco-miR). The results show that high affinities and excellent cellular uptakes can be obtained with all types of arginine-derived PNA, but that differences in anti-miR activity as a function of the type of modification and of the model used are still present, with a contiguous stretch of modified monomers being the preferred model.

Our results do not fully explain the mechanism of action of **PNA4** within the cells. The results obtained are indeed compatible with 1) a decrease in miR-210 processing and maturation, 2) a decrease in miR-210 content due to a possible degradation,^38^ and 3) a decrease in hybridisation availability, as more recently proposed for miR-122.^39^ Further experiments will be required to improve understanding of this issue. Moreover, despite the fact that the activity of **PNA4** appears to be fairly selective, microarray and extensive RT-qPCR analyses will be required to provide a complete view of the effects of **PNA4** on all the miRNA expressed in K562 cells and to pinpoint possible unpredicted off-target events.

Our results might be also relevant in the field of applied biology, because miR-210 has been demonstrated to be linked to hypoxia and up-regulated together with several hypoxia-related genes,^40^ and is hypothesised to be involved in cancer. Because miR-210 is a marker of the growth of advanced tumours under hypoxic conditions and correlates in general with poor prognosis^41^, ^42^ it has been proposed as a possible therapeutic target. Radojicic et al., who analysed 49 primary triple-negative breast cancer cases, along with 34 matched tumour-associated normal samples, for example, reported that miR-210 was overexpressed in triple-negative primary breast cancers, thus suggesting a role in breast cancer tumour progression.^43^ PNAs against miR-210 might therefore be useful on one hand for understanding the function of miR-210 in solid tumours, and on the other to alter miR-210 expression in tumours in which this microRNA has a clear role.

## Experimental Section

**Reagents and instrumentation**: The Boc-protected PNA backbone methyl ester, bearing an l-Arg residue with tosyl protection in the side chain (Boc-5l-Arg_(Tos)_-PNAbackbone-OMe) was prepared from commercial Boc-l-Arg_(Tos)_-OH in three steps, as reported previously.^44^ The corresponding acid (Boc-5l-Arg_(Tos)_-PNA-C_(z)_-OH), the guanine-containing chiral monomer Boc-5l-Arg_(Tos)_-PNA-G_(OBn)_-OH and Boc-2d-Arg_(Tos)_ sub-monomers were obtained by the previously reported procedure.^44^

Reagents and solvents were purchased from commercial sources and used without further purification, except for DMF, which was dried over molecular sieves (4 Å) and purged with nitrogen to avoid the presence of dimethylamine, and THF, which was distilled to avoid the presence of stabilisers. TLC was run on aluminium sheets (Merck 5554 silica 60). Column chromatography was performed as flash chromatography on Merck 9385 silica 60 (0.040–0.063 mm). NMR spectra were obtained with a Bruker AC 300 instrument; *δ* values are in ppm relative to CDCl_3_ (7.29 ppm for proton and 76.9 ppm for carbon) or [D_6_]DMSO (2.50 ppm for proton and 39.5 ppm for carbon). IR spectra were recorded with a Nicolet 5700 FTIR instrument, HPLC-ESI-MS with a Micromass Quattro micro API (QqQ Detector, from 100 % H_2_O to 50 % CH_3_CN in 30 min, 0,2 % formic acid as modifier, flow: 1 mL min^−1^) and HRMS with a Thermo LTQ ORBITRAP XL machine. PNA purification was performed by RP-HPLC with UV detection at 260 nm with use of a semi-prep C18 column (for normal PNA: 5 microns, 250×10 mm, Jupiter Phenomenex, 300 A; for labelled PNA: 10 microns, 300×7.7 mm, Xterra Waters, 300 Å), with elution with water+0.1 % TFA (eluent A) and acetonitrile+0.1 % TFA (eluent B); elution gradient: from 100 % A to 50 % B over 30 min, flow: 4 mL min^−1^.

**Boc-5l-Arg-PNA-T-OMe monomer (1)**: Carboxymethylthymine (190.5 mg, 1.03 mmol) was dissolved in DMF (6 mL) at 0 °C, together with DHBtOH (168.8 mg, 1.03 mmol) and DIPEA (270 μL, 1.55 mmol). EDC**⋅**HCl (198.8 mg, 1.03 mmol) was added and the solution was stirred for 10 min at 0 °C and for 20 min at room temperature; then the Boc-5l-Arg(Tos)-PNAbackbone-OMe (251.2 mg, 0.52 mmol) was added to the mixture. The solution was stirred overnight and the DMF was then removed under reduced pressure. The residue was treated with AcOEt (50 mL) and washed with saturated KHSO_4_ (2×25 mL), saturated NaHCO_3_ (2×25 mL) and brine (25 mL). The organic layer was dried over Na_2_SO_4_ and filtered, the solvent was removed, and the residue was purified by flash chromatography (from AcOEt to AcOEt/MeOH 95:5). Yield: 257.7 mg (76 %); *R*_f_=0.26 (AcOEt/MeOH 9:1); ^1^H NMR (400 MHz, [D_6_]DMSO): *δ*=11.31 (s, 1 H; NH thymine), 7.63 (d, *J*=8 Hz, 2 H; CH tosyl group), 7.29 (d, *J*=8 Hz, 2 H; CH tosyl group), 7.21 (s, 1 H; CH thymine), 7.05 (br s, 1 H; NH guanidinium), 6.83 (d, *J*=8 Hz, 2 H; NH-Boc), 6.79 (br s, 1 H; NH guanidinium), 6.58 (br s, 1 H; NH guanidinium), 4.75 (d, *J*=18 Hz, 1 H; CO-CH_2_-thymine), 4.62 (d, *J*=18 Hz, 1 H; CO-CH_2_-thymine), 4.09 (d, *J*=18 Hz, 1 H; CH_2_ Gly moiety), 3.98 (d, *J*=18 Hz, 1 H; CH_2_ Gly moiety), 3.62 (s, 3 H; OCH_3_), 3.57–3.45 (br m, 1 H; CH arginine), 3.40–3.20 (br m, 2 H; CH_2_ pseudopeptide and water), 3.10–2.95(br m, 2 H; CH_2_NH arginine side chain), 2.35 (s, 3 H; CH_3_ tosyl group), 1.76 (s, 3 H; CH_3_ thymine), 1.55–1.20 (m, 4 H; CH_2_ arginine side chain), 1.36 ppm (s, 9 H; CH_3_ Boc); ^13^C NMR (100 MHz, [D_6_]DMSO): *δ*=169.9, 167.9, 164.8, 157.1, 156.1, 151.4, 142.4, 142.2, 141.5, 129.5, 126.1, 108.6, 78.5, 52.2, 51.9, 48.9, 48.3, 48.1, 29.8, 29.2, 28.7, 21.3, 12.41 ppm; FTIR (KBr): 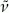
=3444.3 (m), 3342.8 (m, N—H), 3142.8 (w, C—H aromatic), 2978.4 (m), 2958.0 (m), 2929.4 (m, C—H); 1733.7 (s), 1699.9 (s), 1684.0 (s, C—O), 1558.4 (s, N—H), 1472.5 (m, -CH_2_-), 1367.9 (m, S—N), 1253.4 (s, C—N), 1132.5 cm^−1^ (m, S—O); MS (ESI, MeOH): *m*/*z*: 652.3 [C_28_H_42_N_7_O_9_S]^+^, 674.3 [C_28_H_41_NaN_7_O_9_S]^+^, 690.3 [C_28_H_41_KN_7_O_9_S]^+^; HRMS (MeOH): *m*/*z* calcd for C_28_H_41_N_7_O_9_S: 651.26865; found: 652.27661 for [C_28_H_42_N_7_O_9_S]^+^.

**Boc-5l-Arg(Tos)-PNA-T-OH (2)**: A solution of Ba(OH)_2_**⋅**8 H_2_O (175.1 mg, 0.55 mmol) in water (20 mL) was added to a stirred solution of Boc-5l-Arg(Tos)-PNA-T-OMe (239.7 mg, 0.37 mmol) in THF (20 mL). The reaction mixture was stirred for 10 min. The THF was then removed by evaporation and the pH of the solution was lowered to 4.5 with a dilute solution of HCl to induce the precipitation of the product. The solution was cooled at 4 °C for 2 h, filtered (Buchner) and dried under vacuum. Yield: 145.0 mg (62 %); *R*_f_=0.0 (AcOEt/MeOH 9:1); ^1^H NMR (400 MHz, [D_6_]DMSO): *δ*=12.81 (s, 1 H; —COOH), 11.31 (s, 1 H; NH thymine), 7.64 (d, *J*=8 Hz, 2 H; CH tosyl group), 7.29 (d, *J*=8 Hz, 2 H; CH tosyl group), 7.22 (s, 1 H; CH thymine), 7.05 (br s, 1 H; NH guanidinium), 6.93 (br s, 1 H; NH guanidinium), 6.84 (d, *J*=8 Hz, 2 H; NH-Boc), 6.64 (br s, 1 H; NH guanidinium), 4.75 (d, *J*=18 Hz, 1 H; CH_2_ Gly moiety), 4.59 (d, *J*=18 Hz, 1 H; CH_2_ Gly moiety), 4.01 (d, *J*=18 Hz, 1 H; CO-CH_2_-thymine), 3.88 (d, *J*=18 Hz, 1 H; CO-CH_2_-thymine), 3.67 (br m, 1 H; CH arginine), 3.55–2.90 (br m, 4 H; CH_2_NH arginine side chain, CH_2_ pseudopeptide and water), 2.35 (s, 3 H; CH_3_ tosyl group), 1.76 (s, 3 H; CH_3_ thymine), 1.50–1.15 (m, 4 H; CH_2_ arginine side chain), 1.37 ppm (s, 9 H; CH_3_ Boc); ^13^C NMR (100 MHz, [D_6_]DMSO): *δ*=171.4, 170.9, 168.2, 167.7, 164.8, 157.1, 156.1, 151.5, 142.3, 141.5, 129.5, 126.1, 108.6, 78.4, 51.8, 49.0, 48.9, 48.4, 48.13, 29.5, 29.2, 28.7, 21.3, 12.4 ppm; FTIR (KBr): 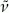
=3437.4 (m, N—H), 3346.0 (m, O—H), 3063.9 (w), 2977.8 (m, C—H), 1683.7 (s, C—O), 1549.6 (s, N—H), 1455.8 (s, -CH_2_-), 1367.8 (m, S—N), 1251.9 (s, C—N), 1132.1 cm^−1^ (m, S—O); MS (ESI, MeOH): *m*/*z*: 638.3 [C_27_H_39_N_7_O_9_S]^+^, 660.3 [C_27_H_38_NaN_7_O_9_S]^+^, 676.2 [C_27_H_38_KN_7_O_9_S]^+^; HRMS (MeOH): *m*/*z* calcd for C_27_H_39_N_7_O_9_S: 637.2539; found: 636.24564 for [C_27_H_38_N_7_O_9_S]^−^.

**PNA oligomer synthesis**: The synthesis of the reference **Pept-1**, **PNA1**, **PNA1-Fl**, **PNA2** and **PNA2-Fl** was reported previously.^25^ The 5l-chiral PNAs were synthesised by standard manual Boc-based chemistry with HBTU/DIPEA coupling; the 2d-chiral PNAs were synthesised by a standard manual/sub-monomeric strategy. All the PNAs were synthesised on MBHA resin loaded with Boc-PNA-G(Z)-OH as first monomer. The fluorescein was introduced by DIC/DhBtOH coupling.

**PNA3**: Yield: 19 %; *t*_r_: 15.9 min; ESI-MS: *m*/*z* found (calcd): 1124.6 (1124.8) [*M*+H_5_]^5+^, 937.4 (937.5) [*M*+H_6_]^6+^, 803.7 (803.7) [*M*+H_7_]^7+^, 703.3 (703.4) [*M*+H_8_]^8+^; *M*_W_ calcd: 5618.3.

**PNA4**: Yield: 11 %; *t*_r_: 16.1 min; ESI-MS: *m*/*z* found (calcd): 1124.9 (1124.8) [*M*+H_5_]^5+^, 937.5 (937.5) [*M*+H_6_]^6+^, 803.6 (803.7) [*M*+H_7_]^7+^, 703.4 (703.4) [*M*+H_8_]^8+^, 625.4 (625.3) [*M*+H_9_]^9+^; *M*_W_ calcd: 5618.3.

**PNA5**: Yield: 3.6 %; *t*_r_: 13.4 min; ESI-MS: *m*/*z* found (calcd): 937.1 (937.5) [*M*+H_6_]^6+^, 803.4 (803.7) [*M*+H_7_]^7+^, 703.2 (703.4) [*M*+H_8_]^8+^, 625.3 (625.3) [*M*+H_9_]^9+^; *M*_W_ calcd: 5618.3.

**PNA6**: Yield: 3.2 %; *t*_r_: 13.4 min; ESI-MS: *m*/*z* found (calcd): 1124.9 (1124.8) [*M*+H_5_]^5+^, 937.5 (937.5) [*M*+H_6_]^6+^, 803.6 (803.7) [*M*+H_7_]^7+^, 703.4 (703.4) [*M*+H_8_]^8+^, 625.4 (625.3) [*M*+H_9_]^9+^; *M*_W_ calcd: 5618.3.

**PNA3-Fl**: Yield: 17 %; *t*_r_: 12.5 min; ESI-MS: *m*/*z* found (calcd): 1224.7 (1225.5) [*M*+H_5_]^5+^, 1020.9 (1021.4) [*M*+H_6_]^6+^, 875.3 (875.6) [*M*+H_7_]^7+^, 766.0 (766.3) [*M*+H_8_]^8+^, 681.0 (681.3) [*M*+H_9_]^9+^, 613.2 (613.2) [*M*+H_10_]^10+^; *M*_W_ calcd: 6122.3.

**PNA4-Fl**: Yield: 17 %; *t*_r_: 12.5 min; ESI-MS: *m*/*z* found (calcd): 1224.8 (1225.5) [*M*+H_5_]^5+^, 1021.0 (1021.4) [*M*+H_6_]^6+^, 875.1 (875.6) [*M*+H_7_]^7+^, 765.9 (766.3) [*M*+H_8_]^8+^, 680.9 (681.3) [*M*+H_9_]^9+^, 613.0 (613.2) [*M*+H_10_]^10+^; *M*_W_ calcd: 6122.3.

**PNA5-Fl**: Yield: 6.8 %; *t*_r_: 12.9 min; ESI-MS: *m*/*z* found (calcd): 1021.1 (1021.4) [*M*+H_6_]^6+^, 875.8 (875.6) [*M*+H_7_]^7+^, 766.3 (766.3) [*M*+H_8_]^8+^, 681.2 (681.3) [*M*+H_9_]^9+^, 613.2 (613.2) [*M*+H_10_]^10+^; *M*_W_ calcd: 6122.3.

**PNA6-Fl**: Yield: 2.8 %; *t*_r_: 12.6 min; ESI-MS: *m*/*z* found (calcd): 1020.8 (1021.4) [*M*+H_6_]^6+^, 875.1 (875.6) [*M*+H_7_]^7+^, 766.2 (766.3) [*M*+H_8_]^8+^, 681.0 (681.3) [*M*+H_9_]^9+^; *M*_W_ calcd: 6122.3.

**Measurements of** *T*_m_ **values**: The *T*_m_ values were determined with a Lambda Bio 20 spectrophotometer and a Peltier PTP6 temperature programmer. Thermal denaturation profiles were measured by monitoring the absorbance at 260 nm from 18 to 90 °C with a heating rate of 1 °C min^−1^ and recording every 0.1 °C. Measurement conditions: [PNA]=[DNA] or [RNA]=5 μm in PBS buffer [pH 7.0, NaCl (100 mm), NaH_2_PO_4_**⋅**H_2_O (10 mm), EDTA (0.1 mm)] with urea (5 m).

**Measurements of circular dichroism spectra**: CD spectra were determined with a Jasco J715 spectropolarimeter and a PTC 348 temperature controller unit. Measurement conditions: strand (5 μm) in PBS buffer (pH 7) at 20 °C.

**Human cell lines and culture conditions**: Human leukaemia K562 cells were cultured under humidified CO_2_ (5 %)/air in RPMI 1640 medium (Sigma) supplemented with foetal bovine serum (FBS, 10 %, Analitical de Mori, Milan, Italy), penicillin (50 units mL^−1^) and streptomycin (50 μg mL^−1^).^26^ Mithramycin (MTH) was from Sigma. Stock solutions of MTH (100 μm) were stored at −20 °C in the dark and diluted immediately before use. Treatment of K562 cells with MTH was carried out by addition of the appropriate drug concentrations at the beginning of the cultures (cells were seeded at 30 000 mL^−1^). The medium was not changed during the induction period. To determine antiproliferative activity, cell growth was studied by determining the cell number per mL with a Z1 Coulter Counter (Coulter Electronics, Hialeah, FL, USA). Erythroid differentiated K562 cells containing haemoglobin were detected by specific reaction with a benzidine/hydrogen peroxide solution as reported elsewhere.^26^ The final concentration of benzidine was 0.2 % in glacial acetic acid (5 m) and H_2_O_2_ (10 %).

**Transfection procedures**: Anti-miR-210 PNAs were directly added to the cellular medium to reach the indicated final concentrations. K562 cells were cultured in 24-well plates starting from the initial density of 30 000 cells per mL. MTH was administrated together with anti-miR PNAs.

**FACS analysis**: For determination of fluorescence intensity by FACScan (Becton Dickinson, Franklin Lakes, NJ, USA), cells (either in medium supplemented with serum or under serum-free conditions) were incubated with the indicated concentrations of PNAs for different lengths of time, harvested and washed. The cells (3×10^5^) were then analysed with the aid of the CellQuest version 3.3 software (Becton Dickinson), with use of the FL1 channel to detect green fluorescence. The results were expressed as median fold (i.e., the ratio between the median fluorescence intensity values obtained in the presence and in the absence of treatment, respectively). A graphic presentation of data was finally obtained through histograms, showing the number of cells versus the expressed fluorescence intensity. In some experiments we pre-incubated fluorescence-labelled PNAs (2 μm) for 10 min at room temperature in the dark with trypsin/EDTA (0.05 %, 2 and 4 μL) before addition to the cells.

**RNA extraction**: Cells were isolated by centrifugation at 1500 rpm for 10 min at 4 °C, washed in PBS and lysed in Tri-reagent (Sigma–Aldrich) according to the manufacturer's instructions. The isolated RNA was washed once with cold ethanol (75 %), dried and dissolved in ultrapure nuclease-free water.

**Real-time quantitative PCR**: For microRNA quantification with real-time RT-PCR reagents, the primers and probes were obtained from Applied Biosystems. Reverse transcriptase (RT) reactions were performed with a TaqMan MicroRNA Reverse Transcription Kit (Applied Biosystems) for hsa-miR-210, hsa-miR-10a, hsa-miR-93, hsa-miR-99b, hsa-miR-126, hsa-miR-149, hsa-miR-424, hsa-miR-483-5p, hsa-miR-625, hsa-let-7c and the iQTM5 real-time PCR System Instrument (Bio-Rad). Relative expression was calculated by the comparative cycle threshold method and U6 microRNA was used as reference gene to normalise all RNA samples, because it is constant in the assayed samples by miR-profiling and quantitative RT-PCR analysis as previously reported.^25^

The nucleotide sequences used for real-time qPCR analysis of γ-globin mRNAs were γ-globin forward primer (5′-TGGCAA GAAGGT GCTGAC TTC-3′), γ-globin reverse primer (5′-TCACTC AGCTGG GCAAAG G-3′) and γ-globin probe (5′-FAM-TGGGAG ATGCCA TAAAGC ACCTGG-TAMRA-3′).^25^ The relative expression was calculated by the comparative cycle threshold method with endogenous control human 18S rRNA as reference genes.^25^
