# Supplementary material for: Cellular Uptakes, Biostabilities and Anti-miR-210 Activities of Chiral Arginine-PNAs in Leukaemic K562 Cells
Source: Chembiochem. 2012 May 25;13(9):1327–37. doi: 10.1002/cbic.201100745 (PMC3401907; doi:10.1002/cbic.201100745)
Supplement: Supplementary file 1 [file cbic0013-1327-SD1.pdf]

CHEMBIOCHEM

Supporting Information

© Copyright Wiley-VCH Verlag GmbH & Co. KGaA, 69451 Weinheim, 2012

Cellular Uptakes, Biostabilities and Anti-miR-210 Activities of Chiral Arginine-PNAs in Leukaemic K562 Cells

Alex Manicardi,[a] Enrica Fabbri,[b, c] Tullia Tedeschi,[a] Stefano Sforza,[a] Nicoletta Bianchi,[b, c] Eleonora Brognara,[b, c] Roberto Gambari,*[b, c] Rosangela Marchelli,[a] and Roberto Corradini*[a]

cbic_201100745_sm_miscellaneous_information.pdf

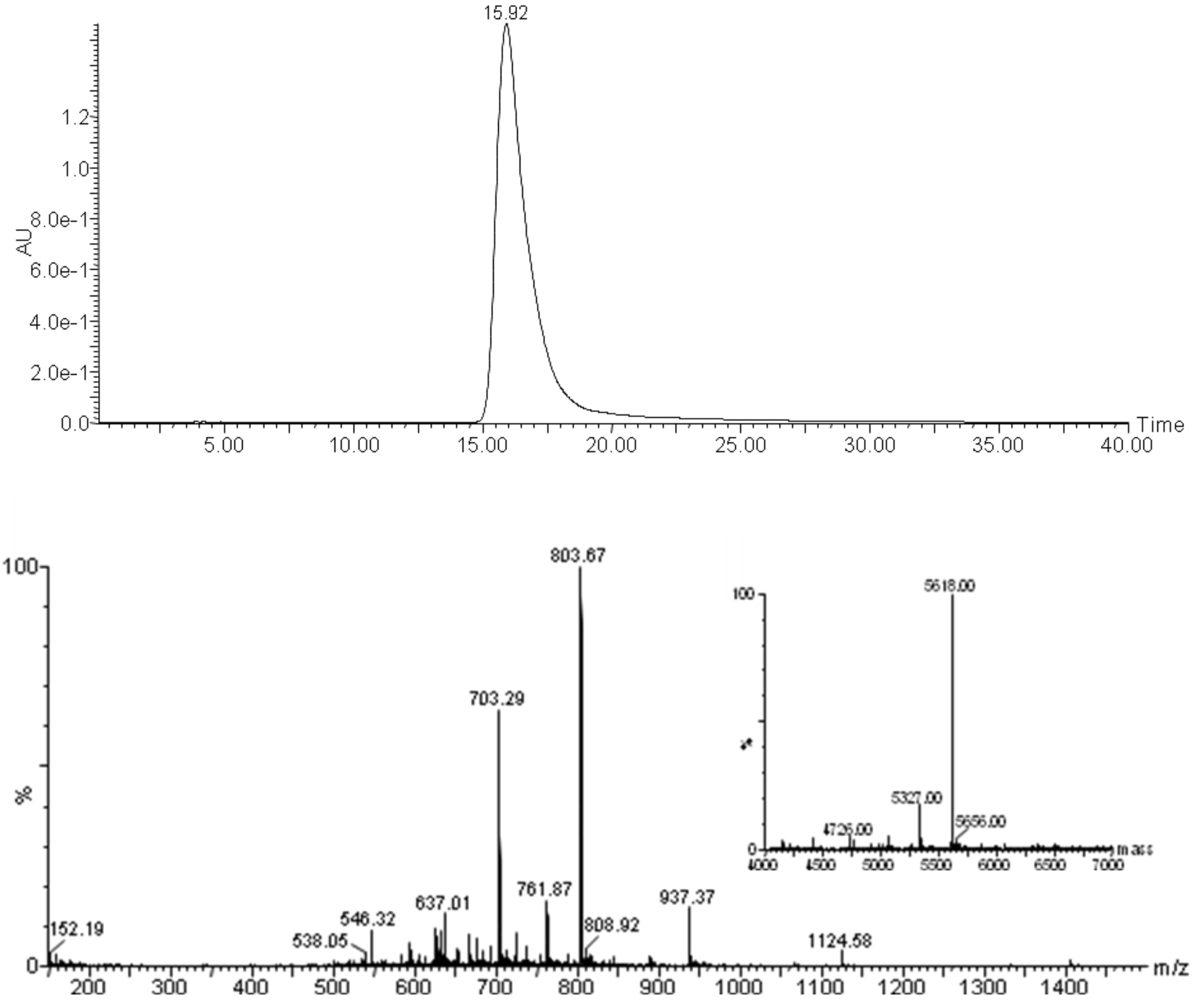

Figure S1. HPLC-DAD trace of **PNA3** measured at 260nm (top), ESI-Q spectrum of the corresponding peak (bottom) and its deconvolution spectrum (insert).

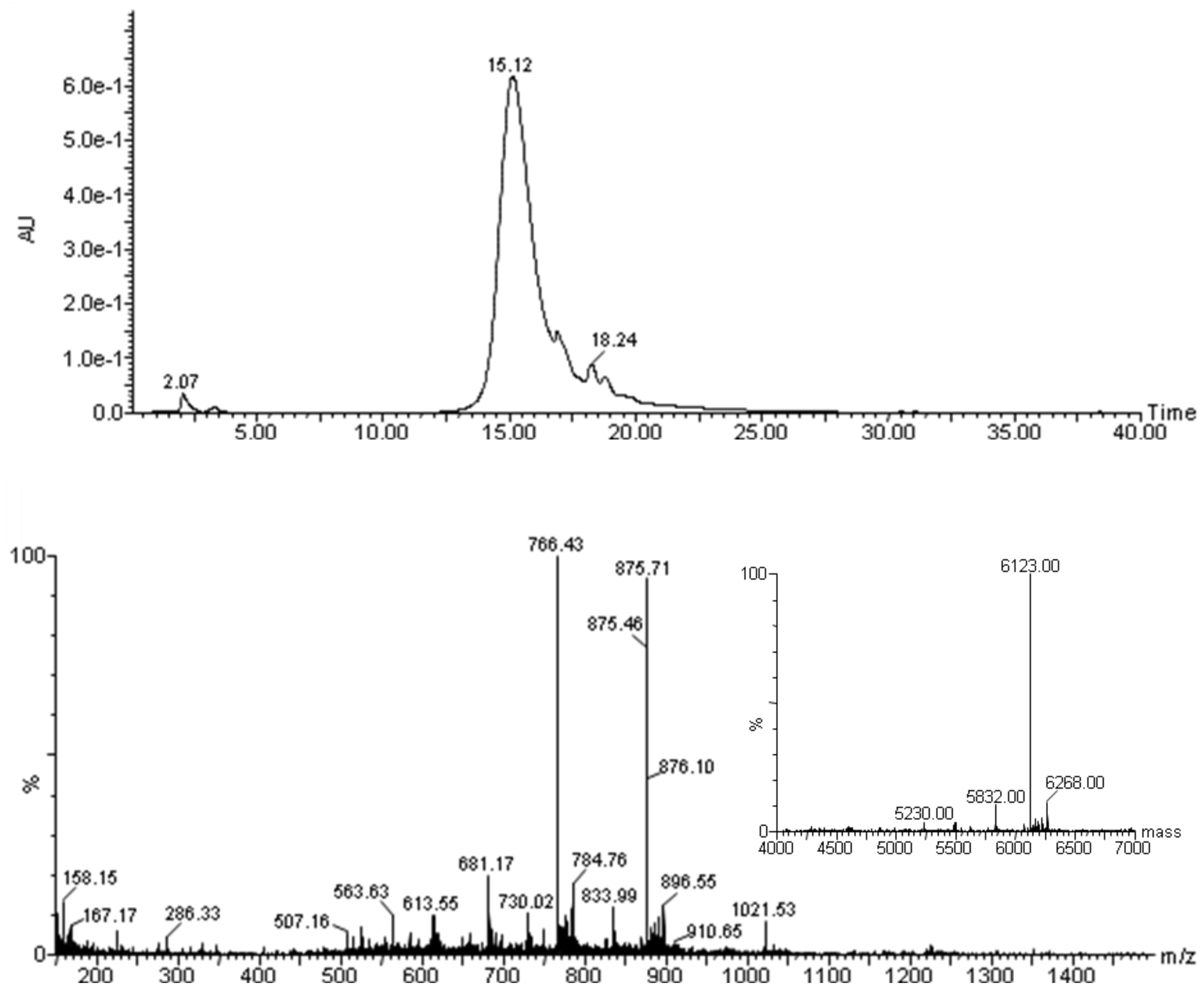

Figure S2. HPLC-DAD trace of **PNA3-Fl** measured at 260nm (top), ESI-Q spectrum of the corresponding peak (bottom) and its deconvolution spectrum (insert).

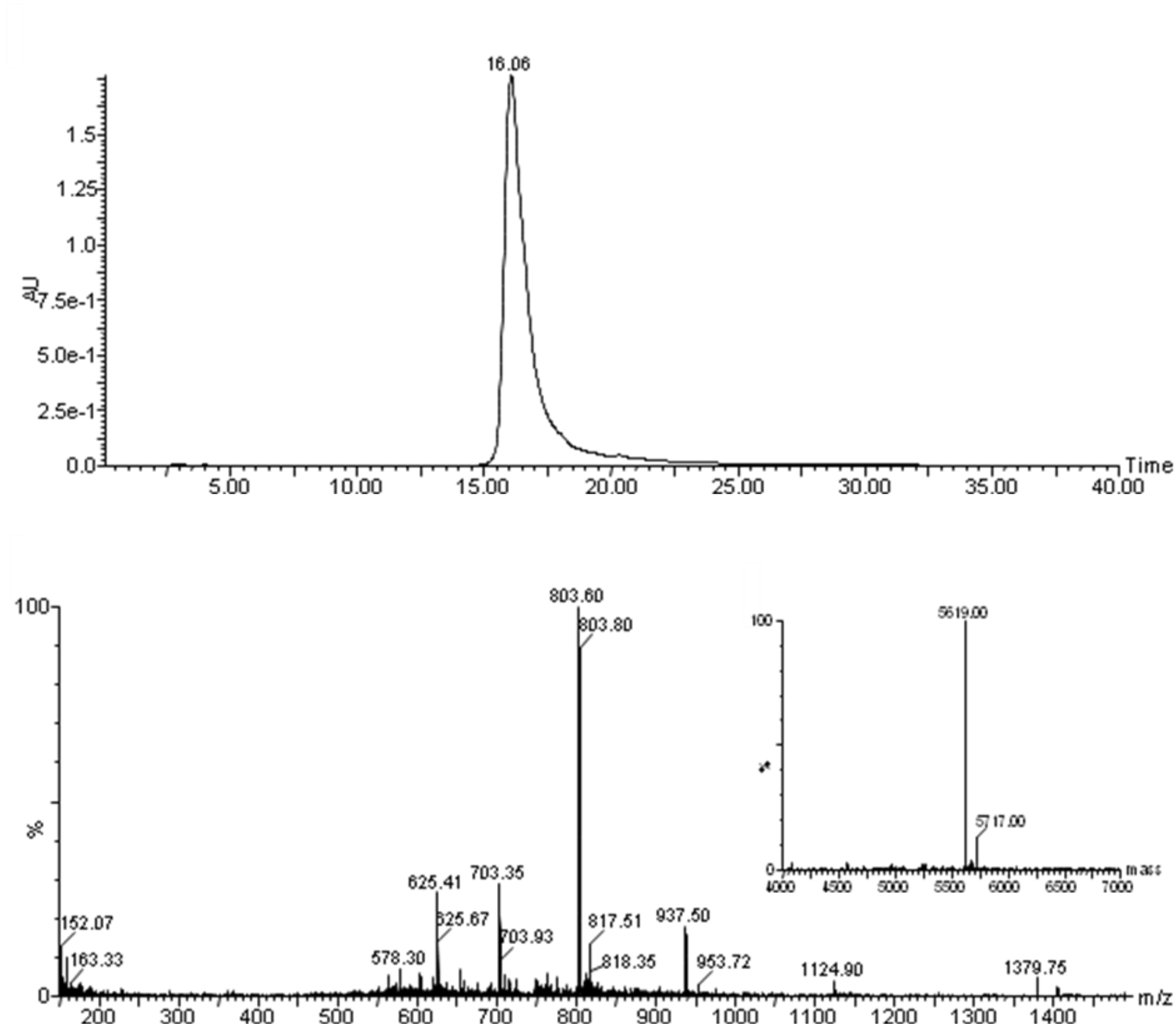

Figure S3. HPLC-DAD trace of **PNA4** measured at 260nm (top), ESI-Q spectrum of the corresponding peak (bottom) and its deconvolution spectra (insert).

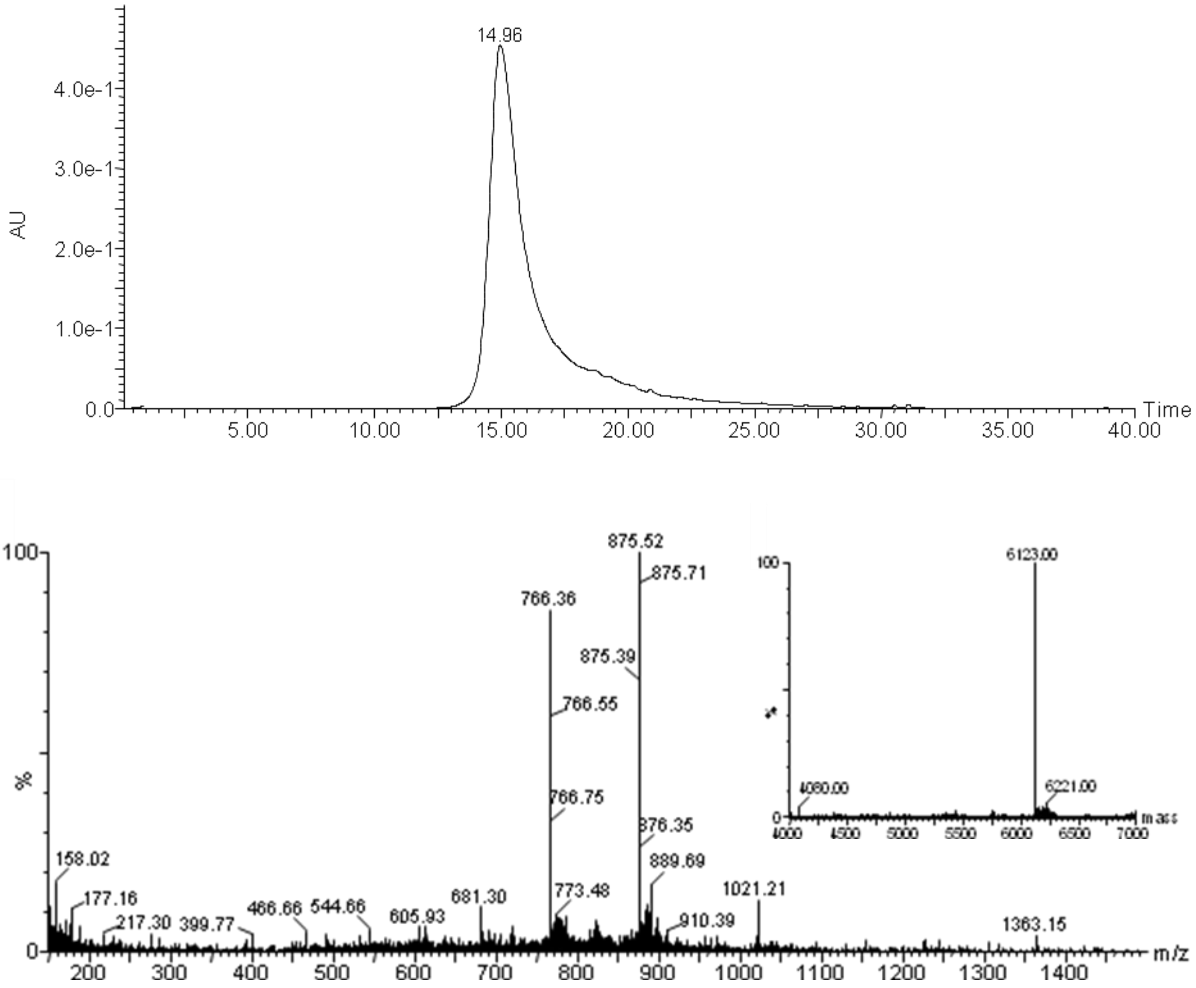

Figure S4. HPLC-DAD trace of **PNA4-Fl** measured at 260nm (top), ESI-Q spectrum of the corresponding peak (bottom) and its deconvolution spectrum (insert).

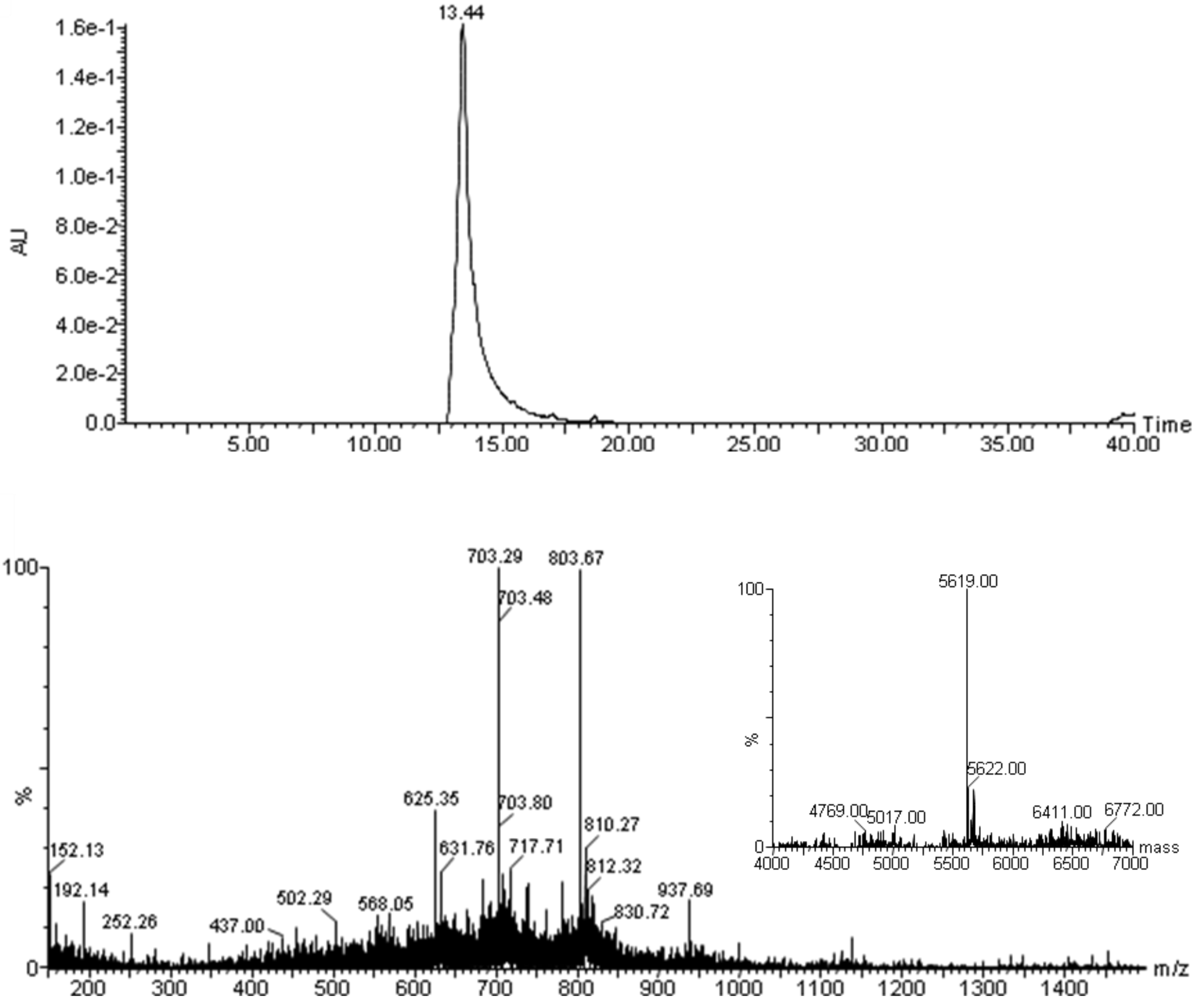

Figure S5. HPLC-DAD trace of **PNA5** measured at 260nm (top), ESI-Q spectra of the corresponding peak (bottom) and its deconvolution spectrum (insert).

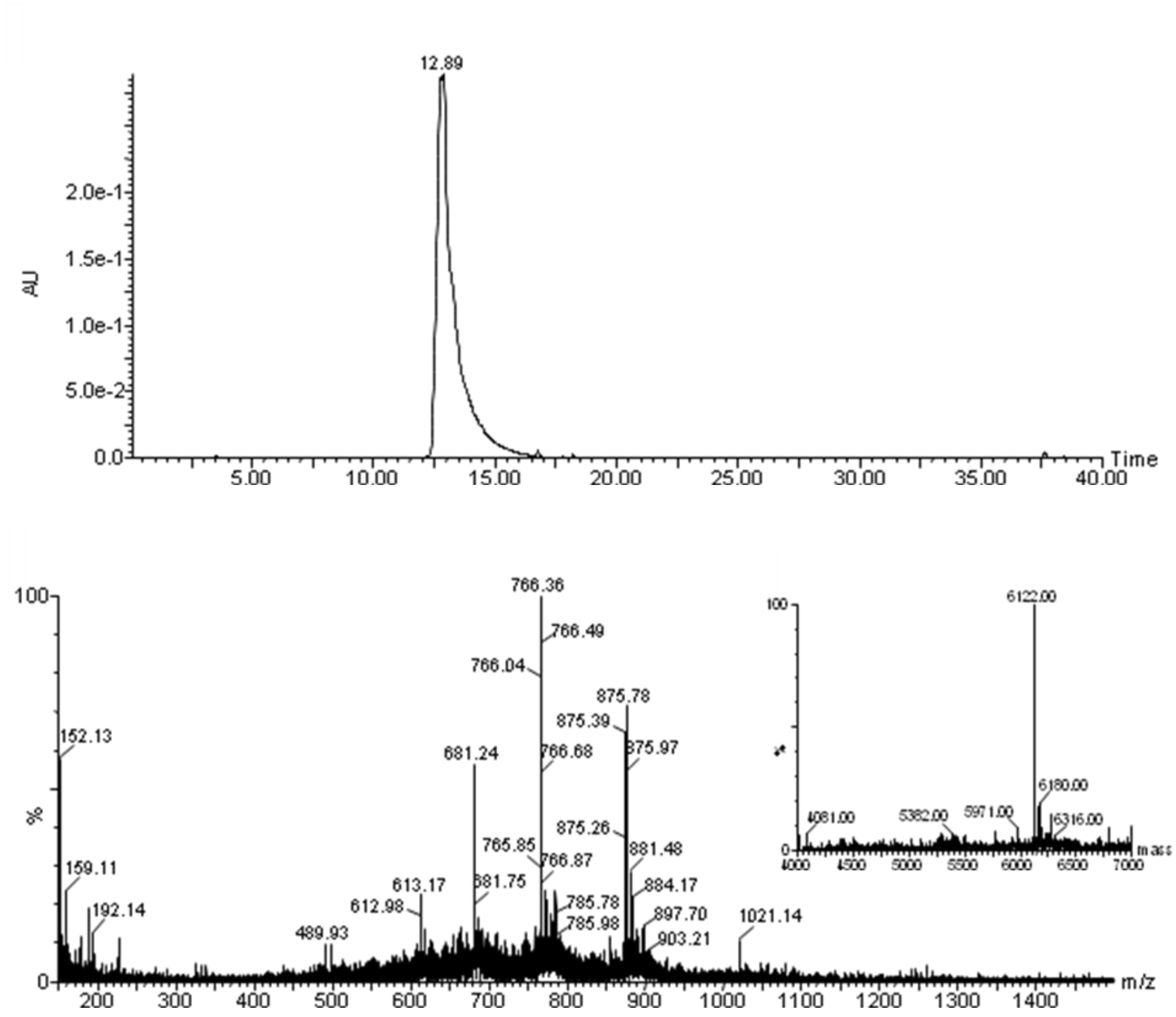

Figure S6. HPLC-DAD trace of **PNA5-Fl** measured at 260nm (top), ESI-Q spectrum of the corresponding peak (bottom) and its deconvolution spectrum (insert).

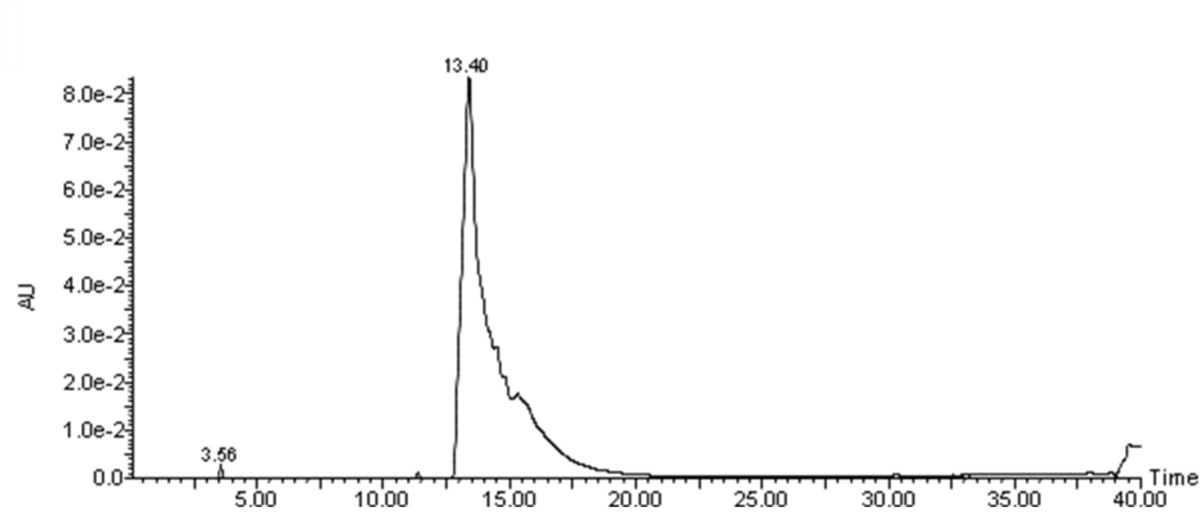

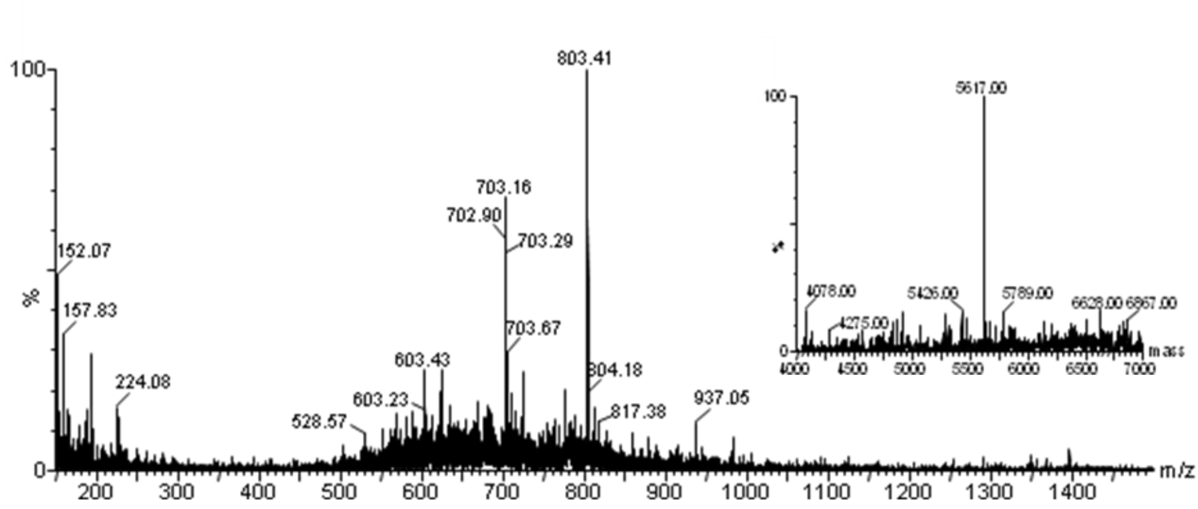

Figure S7. HPLC-DAD trace of **PNA6** measured at 260nm (top), ESI-Q spectrum of the corresponding peak (bottom) and its deconvolution spectrum (insert).

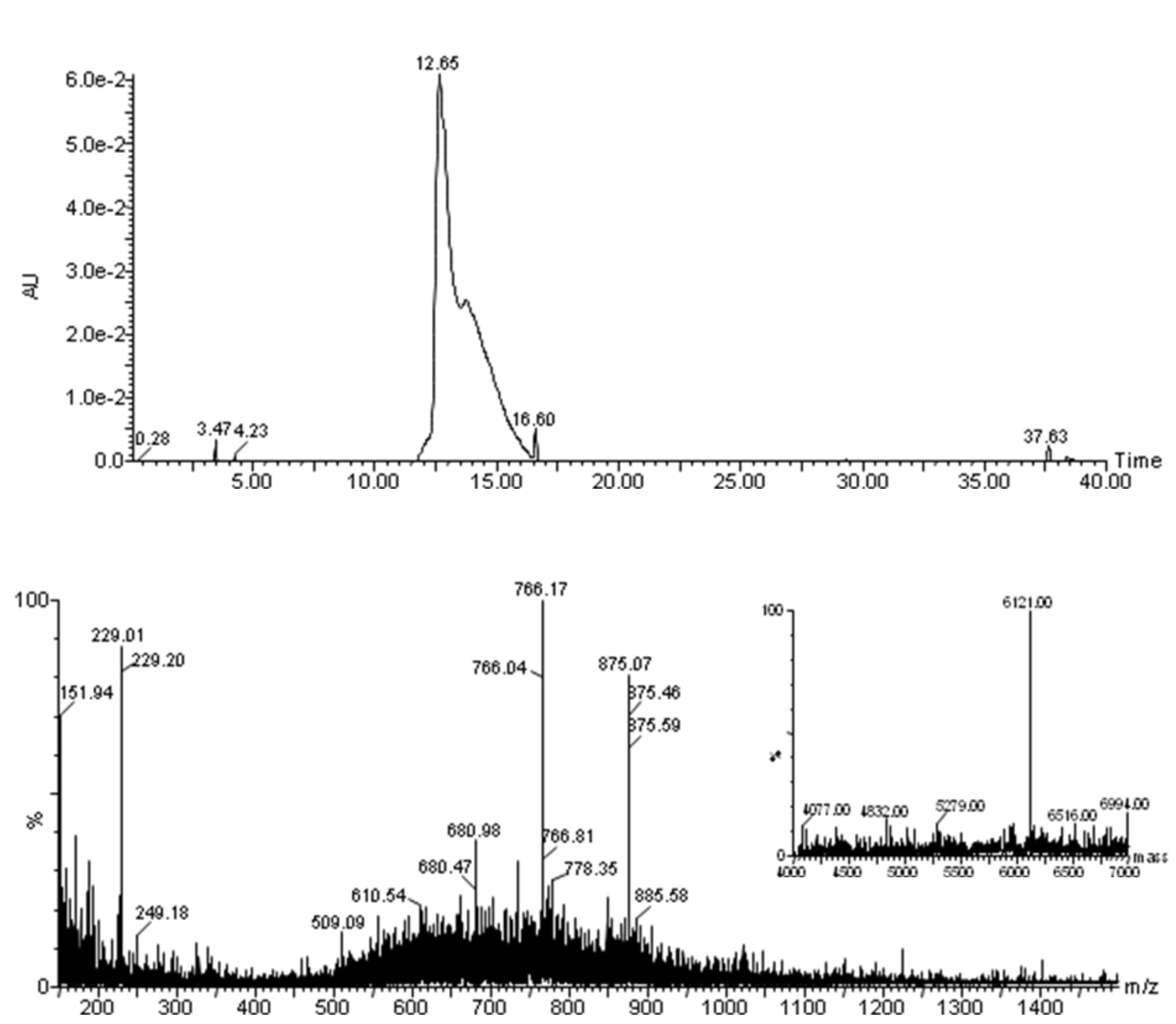

Figure S8. HPLC-DAD trace of **PNA6-Fl** measured at 260nm (top), ESI-Q spectrum of the corresponding peak (bottom) and its deconvolution spectrum (insert).

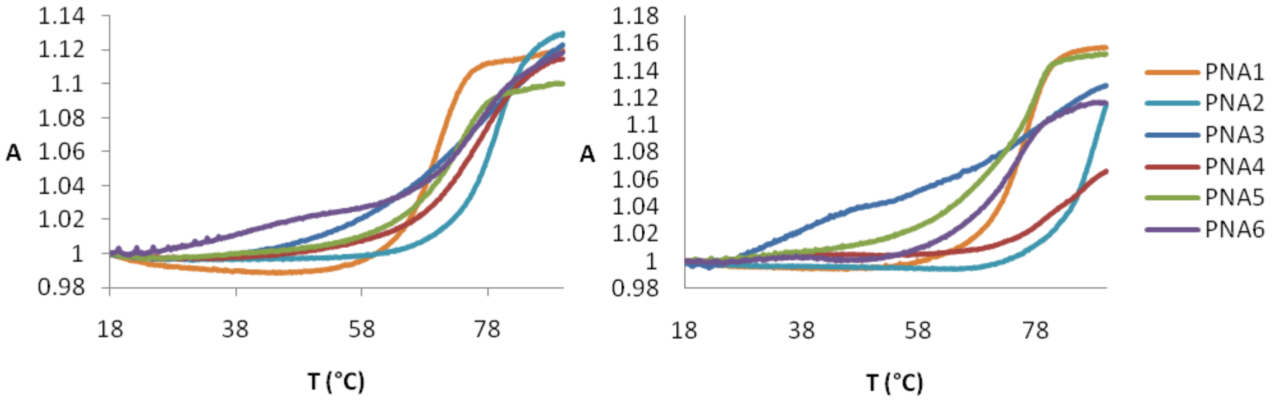

Figure S9. Melting profile of PNA:DNA duplexes (left) and PNA:RNA duplexes (right) measured at 260 nm in PBS pH 7, in presence of 5M urea. Stand concentration: 5 μM.

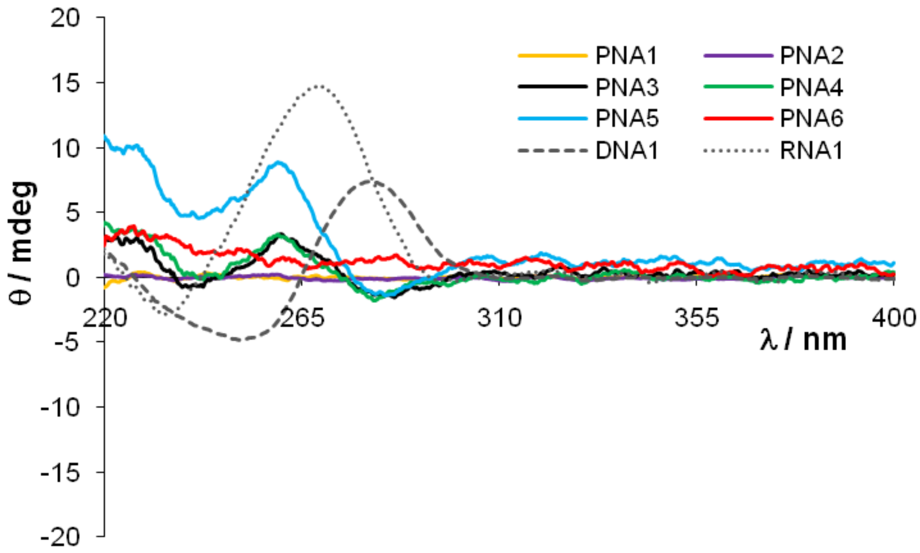

Figure S10. Circular dichroism of ssPNA, ssDNA and ssRNA, measured in PBS pH 7, strand concentration 5 μM.
